# Preanalytical Phase Errors: Experience of a Central Laboratory

**DOI:** 10.7759/cureus.7335

**Published:** 2020-03-20

**Authors:** Cigdem Sonmez, Ummugulsum Yıldız, Nedim Akkaya, Fatma Taneli

**Affiliations:** 1 Clinical Chemistry, University of Health Sciences, Dr. Abdurrahman Yurtarslan Oncology Training and Research Hospital, Ankara, TUR; 2 Clinical Chemistry, University of Health Sciences, Dr. Abdurrahman Yurtaslan Oncology Training and Research Hospital, Ankara, TUR; 3 Clinical Chemistry, Manisa Celal Bayar University, Manisa, TUR

**Keywords:** hemolysis, phlebotomy, preanalytical errors, quality indicator, total testing process

## Abstract

Introduction: The study intends to observe the frequency of preanalytical phase errors both inside and outside the clinical laboratory according to certain quality indicators (QIs).

Methods: The one-week observation focused on 73 nurses drawing blood from 337 patients. It was performed in two stages: the observation of blood collection up to the receipt of the samples, and the receipt of the samples up to the analytical phase. The data pertaining to the number of patients, tests, and rejection rates were obtained from the laboratory information system (LIS) for the one-week and the one-year period and compared with the observational data.

Results: The process of blood sample collection from 337 patients taken into 1347 tubes was observed. Although the majority of the nurses (78%) used safety needles, the safety mechanism was properly activated only in 38% of the interventions. Evaluation of biochemistry tubes (n=971) revealed the following: the incorrect fill volume error was 40%; the hemolysis was seen by 17%, and the clotted sample and fibrin were observed by 6%. The incorrect fill volume error was 12% and 20% in ethylenediaminetetraacetic acid (EDTA) and citrated tubes, respectively. Clotted samples and platelet clumps were seen in 1% of EDTA tubes.

Conclusion: The study confirms the relative frequency of preanalytical phase error occurring inside and outside of the laboratory.

## Introduction

Clinical laboratory testing plays a crucial role in the diagnosis, prevention of disease treatment, and monitoring of patients for better patient care so the performance of high-quality analysis is also critical [1]. In the clinical laboratory testing process, preanalytical, analytical, and postanalytical phases are the three phases of laboratory practice and named as total testing process (TTP) [2-5]. All three phases of the TTP can be dealt with separately, as the analytical errors account for <10% of all errors, and the preanalytical errors account for up to 70% of all laboratory errors [6,7].

The preanalytical phase involves the processes from a physician’s request for a laboratory test request to the preparation of a sample for testing [3]. Patient preparation, sample collection, sample transportation, sample preparation, and sample storage (until the analysis), are the preanalytical steps, which are the major error sources in laboratory diagnostics [3]. Laboratory professionals mainly focused on analytical errors and mistakes in the past. However, laboratory professionals have recently concentrated more on the preanalytical and postanalytical steps due to the increased frequency of the errors [8].

The term preanalytical phase was coined by Statland and Winkel in 1977 and then influence and inference factors were included in the terminology of laboratory medicine [9]. Clinical Laboratory Standard Institute (CLSI) and the World Health Organization (WHO) published quality assurance guidelines for the preanalytical phase. European Federation for Clinical Chemistry and Laboratory Medicine (EFLM) Working Group for Preanalytical Phase (WG-PRE), published an opinion paper in 2015 to ensure effective performance and to increase quality on an international basis in the harmonization of the preanalytical phase of the clinical laboratory [10]. Accreditation programs according to EN ISO 15189:2012 require the laboratory to monitor and evaluate the preanalytical phase using quality indicators (QIs) [11]. Since the preanalytical phase step is mainly performed by the staff working outside the laboratory, it is difficult to manage and evaluate quality in this phase [1,12]. In clinical laboratories, the laboratory staff, especially in the sample reception unit, have to be careful about the written policies for the sample reception and rejection criteria. However, the human factor in the specimen collection and transport procedure are the root cause of these preanalytical phase errors.

This observational study aims to evaluate preanalytical phase activity both outside and inside of our clinical laboratory based on selected QIs and to identify the root causes of preanalytical errors.

## Materials and methods

Study site

This study was conducted in Dr. Abdurrahman Yurtaslan Oncology Education and Research Hospital, Ankara, Turkey, which has 600 beds and was equipped to treat surgery and oncology patients. In addition to routine hospital care services, it includes an intensive care unit (ICU), bone marrow transplantation unit and an emergency department. The Department of Clinical Laboratory of the hospital provides tests for clinical chemistry, clinical microbiology, and hematology tests. The entry of a test request is performed by clinicians in the laboratory information system (LIS). The identification of outpatients is done before blood collection by the secretaries who gave tubes with barcodes to the patients after checking the personal ID numbers. For inpatients, labeling depends on the nurse’s own style; the patient identification is asked before sampling and all the patients have identification labels on their arms. Blood samples are drawn with the guide of LIS in order to use the appropriate tube types and numbers.

Blood samples are obtained at the bedside for the inpatients (at 14 departments and, the emergency department). For outpatients, they are obtained at six different phlebotomy wards. The samples are received or rejected by the staff working in the sample reception unit. In the reception unit, the staff receives or rejects samples by checking the identification, container appropriateness and, filling. If the tubes are proper, they are received and are centrifuged within ten minutes. After centrifugation, the operators re-checked serum and plasma samples visually by grading charts for hemolysis, lipemia. Operators also checked bilirubinemia, clotting, and fibrin visually and, if necessary, they register the rejection of the samples in the LIS.

Study design

Over a one-week period, the preanalytical phase was monitored in two workflow steps. The first step was blood collection and transportation, and the second step was laboratory procedures before the analytical phase. The preanalytical QIs were also assessed. The tube storage conditions, identification of the patient, labeling, disinfection procedures, venipuncture procedures, and safety rules with selected items were checked according to the EFLM WG-PRE Phlebotomy Collection Checklist recommendations for blood collection [11]. All these steps were observed during the blood sample collection by the nurses. The conducted research was not related to either human or animal use.

In the blood sampling period; 14 different departments were observed. The number of observations according to the departments is shown in Table 1. 

**Table 1:** The number of nurses observed during the one-week observation period

Departments	n
Inpatient Hospital Clinical Wards	35 (47.94%)
Urology Clinic	2
General Surgery Clinic	4
Medical Oncology Clinic	8
Post-Operative Intensive Care Unit	4
Bone Marrow Transplantation Center	4
Emergency Department	4
Hematology Clinic	6
Orthopedics Clinic	3
Outpatient Phlebotomy Departments	38 (52.05%)
Ward 1	20
Ward 2	4
Ward 3	6
Ward 4	4
Ward 5	3
Ward 6	1

For inpatients, seven clinical departments and the emergency department were observed. For outpatients, six wards were observed. During the observation period, 73 nurses’ blood drawing from 337 patients into 971 gel tubes (5 mL, BD Vacutainer® SST II Advance, BD-Plymouth UK) was observed. Furthermore, a total of 233 ethylenediaminetetraacetic acid (EDTA) tubes (3 mL, BD Vacutainer ®K2E, 5.4 mg BD-Plymouth UK), and 143 citrated tubes (1.8 mL, BD Vacutainer ® 9NC,0,109 M BD-Plymouth UK) were examined. The samples were transported manually and the mean turnaround time (time interval between sample drawing to laboratory receipt) was 50 minutes. The transport safety, sample reception, and centrifugation conditions were also checked. The temperature of the centrifuge was measured by the external thermometer.

Preanalytical QIs were chosen according to the 2019 IFCC Working Group “Laboratory Errors and Patient Safety” project criteria [13]. The list of rejection criteria referred to in this study is given in Table 2.

**Table 2:** Rejection criteria of the clinical laboratory

Rejection criteria
Misidentification
Inappropriate tubes
Incorrect fill volume
Hemolyzed sample
Clotted sample
Lipemic sample
Icteric sample
Erroneous request

The number of the rejected samples registered in the LIS was also determined for the same observation week so the observation period results could be compared with the LIS data. Also for the comparison of one-week LIS data, the data for a one-year period were also collected from LIS. The one-week and one-year periods overlapped, and they were evaluated in exactly the same way. Rejection rates were calculated by the total number of tubes. As for the erroneous requests, misidentification, improper container or tube use, and incorrect fill volume criteria, the rejection rates were calculated based on the total number of the received samples. In the calculation of the percentage of clotted samples, the following formula was used: number of samples clotted/total number of samples with an anticoagulant checked for clots formula was used. The hemolysis rejection rate was calculated based on the number of samples rejected due to hemolysis and the total number of checked samples for hemolysis. For the calculation of the error rates in the one-week observation period, the following cut-offs were used: for fill volume, <90%; for hemolysis, (+) in the visual hemolysis chart. The acceptable filling volume of the citrated tubes was ± 10% of that marked on the tube. Visual hemolysis grading chart‘s (+) hemolysis corresponds to >2g/L free hemoglobin.

Statistical analysis

One-week observation period data and one-year test number data were obtained from the LIS system. Statistical analyses were performed by the Statistical Package for the Social Sciences (SPSS) version 20.0 (SPSS Inc., Chicago, IL) program. The observation period and LIS data were compared by student t- test and p <0.05 was considered statistically significant.

## Results

Phlebotomies observed during the study were mainly performed by nurses. During the observation period, 337 blood samples were drawn into 1347 tubes by 73 nurses. When the consumables were checked, it was found that three clinics were using expired citrate tubes (only six tubes), while all the other consumables were stored in appropriate conditions. One of the most frequent errors that were observed was the patient identification error (78%), especially at the outpatient phlebotomy wards. The labeling of the tubes was done before blood collection (80%).

The majority of nurses put on clean gloves prior to the blood sampling procedure (92%), but they did not perform the correct disinfection procedures in the collection of blood sampling (90%). Single-use of the holder and the tourniquet were seen only in 3% and 8% of the blood drawing procedures, respectively. Tourniquet was placed correctly in 99% of the blood drawing. Venipuncture sites displayed the following: 288 on the antecubital fossa, 39 on hand, and 25 by the catheter. For blood drawing, the nurses used 263 needles (BD- sterile®, Plymouth, UK) with a safety mechanism, 26 conventional needles with the holder, 38 injectors, and 25 catheters with uerlock. Although the safety devices were used in 78% of the blood drawing, correct activation of the safety mechanism was performed only by 38%. However, the correct device disposal was observed in 36% of them. Appropriate tourniquet time and release were not applied in 84% of the procedures.

The order of citrated and EDTA tubes was wrong in 5% and 75% of the drawings, respectively. EDTA tubes were collected before gel tubes. Sample tubes were not immediately and appropriately mixed with the inversion (5-6 times) in 94% of the tubes by nurses. Specimens were transported manually by ward staff, and turnaround time (TAT) was approximately 50 minutes (42-63 minutes). The centrifuges temperature was very high (44.2 Celcius for gel tubes; 43.7 Celcius for citrated tubes centrifugation).

The fill volume, hemolysis, and clot were evaluated in the one-week observation period.The fill volume error was seen in 40% of gel tubes (n= 387); in 242 of the gel tubes the fill volume was <75%, and in 145 of the gel tubes the fill volume was <50%. The hemolysis was observed in 165 of the gel tubes (145, trace and 20, (+) hemolysis by the grading chart). Fibrin was seen in six of the gel tubes. The numbers of the tubes with EDTA and citrate that have fill volume errors were both 28. Fourteen of the tubes with EDTA had fill volume <75%, whereas the remaining 14 tubes fill volume was <50%. Clotted samples and platelet clumps were seen in 1% of EDTA tubes. Only one citrated tube was overfilled (above the acceptable limit), whereas the 27 citrated tubes had fill volume <90%. Hemolysis was seen in 15 tubes of the citrate tubes; nine had trace and six had trace (+) hemolysis. Hemolysis occurred especially in the tubes collected by the syringe and catheter route.The results of the phlebotomy observation period survey are shown in Table 3. 

**Table 3:** Phlebotomy observation period survey CLSI: Clinical Laboratory Standard Institute.

Number	Question	Yes	No
1	Did the collector assemble all necessary supplies prior to collection?	100	0
2	Did the collector check the expiry dates of devices in use? (6 citrated tube)	99.5	0.5
3	Did the collector identify the patient according to CLSI or local guidelines	22	78
4	Did the collector put a new fresh clean pair of gloves	92	8
5	Did the collector appropriately sanitize hands?	10	90
6	Did the collector place the tourniquet 4 finger-widths (10cm) above the venipuncture site?	99	1
7	Did the collector select a suitable venipuncture site according to standard	89	11
8	Did the collector clean the venipuncture site?	100	0
9	Did the collector release the tourniquet when blood flow commenced?	16	84
10	Was the collector using a closed system for venipuncture?	93	7
11	Did the collector follow the correct order of draw according to the guidelines? (for citrate tubes)	95	5
12	Did the collector follow the correct order of draw according to the guidelines? (for EDTA tubes)	25	75
13	Were any of the sample tubes clearly under or over filled?	33	67
14	Were all sample tubes immediately and appropriately mixed according to manufacturers’ specifications?	6	94
15	Did the collector place a clean gauze or cotton ball over the venipuncture site?	100	0
16	Was the safety feature in the blood collection system activated immediately?	38	62
17	Was the needle/collection system safely and immediately disposed?	36	64
18	When were the sample tubes labeled? (Pre-Post)	80	20
19	Was the collection successful i.e. all required tubes collected from a single venipuncture?	95.7	4.3

The total number of tubes, the number of errors, and the rejections rates are shown in Table 4. For the hemolyzed sample, the incorrect fill volume and the clotted sample error type’s p-values were <0.05, while for the misidentification error, inappropriate tube and erroneous request significance value were >0.05. For the one- week observation period, the wrong draw order was seen in 13.5% of the tubes, and it was not possible to examine it in the routine laboratory period. Also, the wrong request (e.g. Prothrombin Time (PT) mixing test requested before PT test) could only be monitored from LIS and was seen by 0.11% and 0.01% in one-week and one-year LIS periods.

**Table 4:** Rejection rates according to the rejection criteria of the clinical laboratory at one-week observation period, one-week and one-year LIS period LIS: laboratory information system; n: number; a: p value <0.05. Rejection rates were calculated by the total number of the tubes that were accepted during the dedicated time interval with the following formula: number of rejected samples/total numbers of samples × 100. For the erroneous request, misidentification, improper container or tube, and incorrect fill volume criteria, rejection rates were calculated with the total number of the tubes that were accepted. For clotted samples we used the total number of samples with an anticoagulant checked for clots whereas the calculation of hemolysis rejection rate was done with the number of samples rejected due to hemolysis per total number of checked samples for hemolysis.

	One-week LIS Period			One-year LIS Period			One-week Observation Period		
Error Type	Error (n)	Total tube n	%	Error n	Total tube n	%	Error n	Total tube n	%
Misidentification error	0	3376	0	64	472433	0.01	0	1347	0
Inappropriate tube	1	3376	0.03	161	472433	0.03	0	0	0
Incorrect fill volume	5	3376	0.14	157	472433	0.03	201	1347	14,9 _a_
Hemolyzed sample	0	1525	0	486	202085	0.24	26	1116	2,24_a_
Clotted sample	8	576	1.55_a_	767	232792	0.32	2	376	0.53
Erroneous request	4	3376	0.11	78	472433	0.01	N/A	N/A	N/A

## Discussion

Maximizing the clinical laboratory quality process is gaining increasing importance, and identifying preanalytical errors correctly is becoming critical at each step of this phase. Preanalytical error types especially occur outside the laboratory, and the root cause of the error is human [4,14]. A study of Romero et al., which consists of two tertiary hospital laboratories, also reported that the preanalytical phase potential errors were mainly detected outside the laboratory facilities [15].

In the observation period of the study, the identification of the patient and the labeling of the tubes were found improper according to the EFLM WG-PRE recommendations. Similarly, Carraro et al. observed high rates of patient identification errors at the time of blood sampling, similar to the results of the present study [16]. In another study, Carraro and Plebani shared their ten years of experience in a Stat laboratory, wherein they pointed out that the patient identification error (8.8%) was the second-highest error types [8]. Kadic et al. showed that the proportion of identification errors was eight fold higher in the outpatient (collection sites outside the hospital) than in the inpatient samples [17]. It was observed that the identification of the patient which seemed to be erroneous in the present study, was performed by the secretaries rather than nurses before blood drawing. Training programs were performed repeatedly to the medical secretaries to avoid patient identification errors.

Although the blood drawing procedure had some correct steps such as the use of a single safety needle, overall the safety procedures were not appropriately performed. Similarly, the venipuncture site area was appropriate, while the tourniquet time was not. In the observation period, the order of drawing according to the tube type was found to be wrong, and the tube fill volume was ignored by the nurses, while the coagulation and EDTA tube fill volume errors were lower than the serum tubes. Also after drawing, mixing was not performed properly by the nurses; the mixing cycle was inadequate. Simundic et al. showed that the total error was 26% (10.6-43.8) in the phlebotomy procedure in their observation study, which was performed in compliance with the CLSI H3-A6 guidelines [11]. Similar to our results, the most correct step of the phlebotomy was the venipuncture site collection [11]. Another similarity with the study of Sumindic et al. was related to the incorrect order of drawing [11]. The observation findings revealed the inadequate education and training procedure of the nurses in the study group and showed that regular education programs must be implemented to them. Lillo et al. showed that educational programs for nursing personnel dramatically decreased the preanalytical sample errors [18]. Makhumula‐Nkhoma et al. also published that the importance of continued education and training and monitoring and evaluating the hemolysis rate is crucial [19]. Romero et al. stated that they prioritized the potential problems in the preanalytical phase with the use of Healthcare Failure Mode and Effects Analysis (HFMEA), and then to improve their quality, they mainly focused on a specific training program and revision of the existing protocols [15].

In the preliminary study, when the rejection types and rates were checked, in the one-week observation period especially, the fill volume error, hemolysis, and clotting, and rejection rates were found higher than the LIS data. The LIS data for one week and one year had similar types and rates. When evaluated in the one-week observation period the rejection rates for fill volume, clotting, and hemolysis were 14.9%, 0.53%, and 2.24%, respectively. The observation period fill volume error had the highest percentage because both the nurses and the laboratory sample reception department staff checked especially for the EDTA and citrate samples’ fill volume, while they paid less attention to gel tubes. Carraro et al. observed the pre-preanalytical period in three wards and found no significant error rate differences between the observation period and the overall six month study period [16]. The present study shows the lack of comparability of the observation period and LIS data. The fill volume error and hemolysis error rates were higher in the observation period except for the clotted sample error. This may be attributed to the habitual procedures applied by the staff and the nurses in the preanalytical phase and failure to implement the written quality procedures. Carraro et al. study, fill volume error was the highest, especially in citrate tubes as in the present study [16]. Their other error types were hemolysis and incorrect sample shipping, (62 tubes 1148 ppm) and clotting [16].

The analysis of clotting error rates revealed contradictory findings. As for clotting error type, one-week LIS data (1.55%) was the highest, and the one-year LIS data (0.32%) was the lowest. This might be random, or can be linked to the increased awareness in the one-week period due to the presence of observation. In contrast, the hemolysis rate in the one-year LIS period was lower than the one-week observation period. The hemolysis was not observed in the one-week LIS period.

When the one-year LIS period rejection rates were evaluated, the clotted sample was found to be the most common rejection criteria. Atay et al. reported that the rejection rates for hemolysis, clotted specimen, and insufficient volume were 8%, 24%, and 34%, respectively whereas the fill volume error had the highest rate [14]. In another study, they mostly observed the fill volume error type [8]. These studies did not confirm our data about the preanalytical error types. However, other previously published studies in the literature reported that their rejection error types were mostly due to the hemolyzed and clotted sample [17,20,21]. All of these findings were in compliance with the error types found in this study. 

The limitation of this study was the short duration of the observation period and the limited number of observed nurses.

## Conclusions

The present study confirms the relative frequency of preanalytical phase errors outside and inside the laboratory. This study emphasizes the importance of the nurse and laboratory staff training during the preanalytical phase and showed that periodic auditing is essential for implementing the existing procedures. The study has practical implications for our laboratory; corrective strategies to improve the process should be introduced. For example, the plan-do-check-act cycle (PDCA) (Deming cycle, Shewhart cycle) should be implemented immediately to ensure continuous quality improvement. Also, guidelines for phlebotomy and centrifuge procedures can be written in the mother tongue and used for training process. It is more likely that guidelines prepared in the mother tongue will increase comprehension and decrease possible procedural errors that stem form information gap. Still, it draws attention to the fact that implementation of QIs, especially in the preanalytical phase, is crucial to achieving reliable results from laboratories which have a very important effect on patient safety and on patient’s clinical outcomes.
